# Information flow dynamics between geopolitical risk and major asset returns

**DOI:** 10.1371/journal.pone.0284811

**Published:** 2023-04-25

**Authors:** Zaghum Umar, Ahmed Bossman, Sun-Yong Choi, Xuan Vinh Vo

**Affiliations:** 1 College of Business, Zayed University, Abu Dhabi, United Arab Emirates; 2 Department of Finance, University of Cape Coast, Cape Coast, Ghana; 3 Department of Financial Mathematics, Gachon University, Seongnam, Republic of Korea; 4 Institute of Business Research, University of Economics Ho Chi Minh City, Ho Chi Minh City, Vietnam; Massey University - Albany Campus: Massey University - Auckland Campus, NEW ZEALAND

## Abstract

We quantify information flows between geopolitical risk (GPR) and global financial assets such as equity, bonds, and commodities, with a focus on the Russian-Ukrainian conflict. We combine transfer entropy and the I-CEEMDAN framework to measure information flows at multi-term scales. Our empirical results indicate that (i) in the short term, crude oil and Russian equity show opposite responses to GPR; (ii) in the medium and long term, GPR information increases the risk in the financial market; and (iii) the efficiency of the financial asset markets can be confirmed on a long-term scale. These findings have important implications for market participants, such as investors, portfolio managers, and policymakers.

## Introduction

As globalization accelerates, the flow of information among global financial markets has increased. Information flow has been crucially considered in both academic and professional circles, as it relates to efficient market hypothesis and portfolio diversification [1, 2]. Furthermore, as several financial crises strengthened the integration of financial markets, information flow between financial markets became more important [3–5].

The global financial markets have been undergoing great turmoil recently due to the Russian-Ukrainian conflict. As major countries impose strong economic sanctions on Russia, the global financial markets have been directly affected. In particular, this occurs post-COVID-19, causing major disruptions to the global supply chain(See, ‘Another year of crazy hiccups’: Russia and China pose new threats to global supply chain, The Washington Post, https://www.washingtonpost.com/business/2022/03/18/russia-ukraine-war-china-covid-supply-chain). Consequently, the Russian-Ukrainian conflict caused great turmoil in domestic, regional, and international financial markets. In other words, relationships among global financial assets have been altered by geopolitical risk.

In this study, we see how the geopolitical risk due to the Russian-Ukrainian conflict affects the financial markets and examine information flows between geopolitical risk and global financial assets. To do this, we employ the GPR index introduced by [6] and various financial assets (Russian bonds, European bonds, Russian equity, European equity, crude oil, gold, natural gas, and wheat). We analyze the impact of the conflict on global financial markets, and our findings provide important implications for both investors and policymakers, for instance, on portfolio diversification.

To measure information flows between the GPR index and global financial markets, we employ the improved complete ensemble empirical mode decomposition with adaptive noise (I-CEEMDAN) method proposed by [7]. The I-CEEMDAN method has been used to forecast time-series data in various fields [8–11]. It has recently been employed to examine information flows in financial markets [12, 13]. In addition, several studies employing the transfer entropy to study information flows in financial markets exist. First, there are a few studies that examine information transmission between individual stocks and stock markets [14–16]. Second, there are additional studies on information flows between various financial markets, such as stock, commodities, foreign exchange, bond, and credit markets [17–19]. In addition, there are also studies on information flow between cryptocurrencies [20] as well as information flow between economic policy uncertainty and stock markets [21].

This study adds to the existing literature in the following ways: First, we analyze the impact of the Russian-Ukrainian conflict on the global financial markets in terms of information flow, which, to our knowledge, has not been addressed in earlier studies. Specifically, we focus on the information flow from the GPR index to financial assets; this has important implications for market participants. Second, we decompose the return series using the I-CEEMDAN method and employ the Rényi transfer entropy to measure the information flow between the GPR index and financial asset returns. The I-CEEMDAN is a useful data-driven model that can be used to explain nonlinear and complex data [22, 23]. Furthermore, the Rényi transfer entropy has the advantage of being flexible when it comes to measuring information flows [20]. However, only a few studies have applied the I-CEEMDAN method to financial market data; our results fill in these gaps in the literature.

It is noteworthy that in the spirit of the fractal market hypothesis, as espoused by [24], market participants’ behavior is unevenly spread across trading horizons, which we can infer from multi-scales. So, as markets respond rapidly to evolving events, investors also modify their risk-return preferences to suit the prevailing condition [25]. Therefore, market dynamics are likely to be heterogeneous across trading horizons. For this reason, it is informative to analyze the dynamics of information transfer from geopolitical risk shocks to different assets across various investment scales classified into the short-, medium-, and long-term horizons. Similarly, due to the fat-tailed attribute of financial time series, which amplifies in Black Swan periods [13, 26], it is important that the behavior of financial markets towards shocks is analyzed across multiple scales, which is achieved by segregating the original time series into their respective scale components. To account for these factors, the application of econometric approaches that cater for the heterogeneous and complex attributes of the market is necessary.

The existing literature on the impact of the Russian-Ukrainian geopolitical risk on financial markets largely covers methods like the event study approach [27], spillovers [28], wavelet [29, 30], and quantile regressions [4, 31]. None of these works have yet explored the dynamics of information flow from geopolitical risk shocks to financial markets despite the fact that information flow between is intensified in stressed periods of financial markets. Therefore, we extend the existing evidence on the degree of effect of the Russian-Ukrainian geopolitical risk on various assets. The achievement of this is facilitated by the transfer entropy analysis grounded on the information flow theory. We expect that if various financial markets are efficient, then, they should be affected similarly by the geopolitical risk shock. If there are variations in the patterns of information flow, we can infer diversification attributes. Thus, our transfer entropy-based analysis caters important implications for risk management in times of intense geopolitical risk (GPR). We find that, in the short term, crude oil and Russian equity show opposite responses to GPR; in the medium and long term, GPR information increases the risk in the financial market; and the efficiency of the financial asset markets can be confirmed on a long-term scale where various assets tend to bear similar responses to GPR. These findings are relevant for managing portfolios across different investment horizons.

The remainder of this paper is organized as follows. Section 2 briefly describes the data and the methods used in this study. The study’s empirical findings are presented in Section 3. Finally, in Section 4, we make some concluding remarks.

## Data and methods

### Data

**Fig 1:**
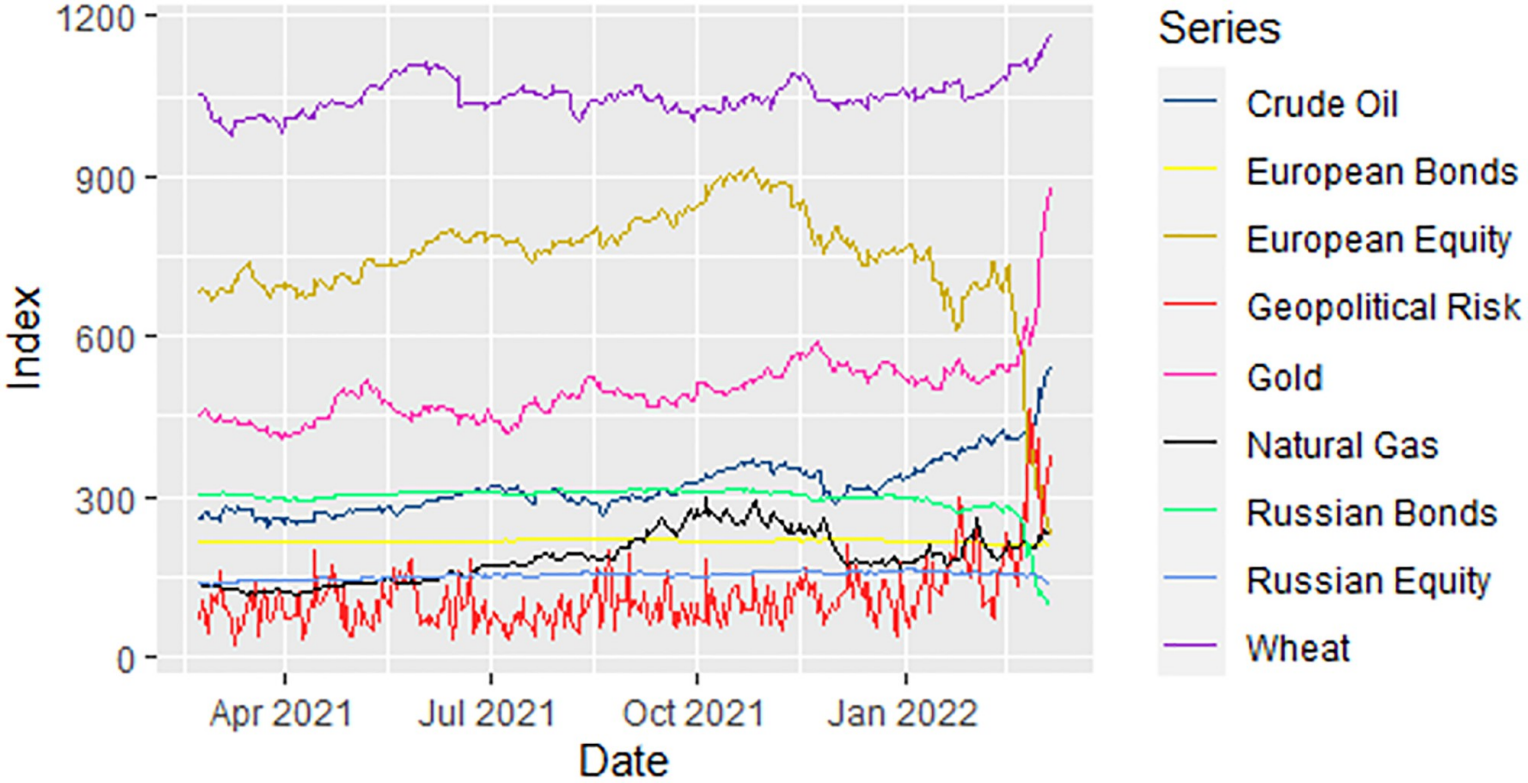
Trajectories of asset and geopolitical risk series (February 22, 2021, to March 7, 2022).

We utilize the daily log-return series of Russian bonds, European bonds, Russian equity, European equity, crude oil, gold, natural gas, and wheat, in addition to daily geopolitical risk. The sample period spans from February 22, 2021, to March 7, 2022, and the trajectories of the raw series are shown in Fig 1. The start date is chosen to mark a day after the announcement by the Russian Defense Ministry on February 21, 2021, regarding the deployment of paratroopers at the borders of Ukraine.

A descriptive summary and a correlation matrix of the return series are presented in Table 1. It is clear from the table that all the return series have a mean value close to zero and do not follow a normal distribution. Among financial assets, the return series of Russian bonds and European equity show large fluctuations between their minimum and maximum values. Furthermore, among the assets, the return series of Russian bonds and European equity is the most left-skewed and has the largest kurtosis values. These results imply that the returns on these two assets were the most affected by the Russian-Ukraine conflict. Additionally, in the correlation matrix, the return on the GPR index is predominantly negatively correlated with financial asset returns but positively correlated with crude oil and Russian equity.

**Table 1:** Summary statistics.

Panel A: Sample statistics									
	Russian bonds	European bonds	Russian equity	European equity	Oil	Natural gas	Gold	Wheat	GPRD
Minimum	-0.40	-0.01	-0.04	-0.48	-0.14	-0.12	-0.08	-0.05	-1.43
Maximum	0.11	0.02	0.03	0.23	0.08	0.15	0.08	0.02	2.49
Average	0.00	0.00	0.00	0.00	0.00	0.00	0.00	0.00	0.15
Std.dev	0.03	0.00	0.01	0.05	0.02	0.04	0.02	0.01	0.55
Skewness	-8.17	0.88	-0.92	-5.45	-0.94	-0.25	0.56	-0.81	0.24
Kurtosis	81.48	6.28	3.08	54.51	5.93	1.84	1.90	3.33	0.88
Normtest.W	0.26***	0.94***	0.93***	0.47***	0.93***	0.96***	0.96***	0.94***	0.99*
Panel B: Correlation matrix									
Russian bonds	1.00								
European bonds	-0.24	1.00							
Russian equity	0.32	-0.20	1.00						
European equity	0.86	-0.20	0.48	1.00					
Oil	-0.21	-0.12	0.18	-0.07	1.00				
Natural gas	-0.02	0.14	-0.07	0.03	0.04	1.00			
Gold	-0.42	0.04	-0.19	-0.36	0.23	-0.02	1.00		
Wheat	-0.15	0.28	-0.03	-0.11	0.10	0.09	0.28	1.00	
GPRD	-0.05	-0.01	0.06	-0.02	0.01	-0.03	-0.02	-0.04	1.00

Notes: GPRD is daily returns of geopolitical risk indices; Normtest.W is the statistic of a Shapiro-Wilk test of normality;

*[10%] and

***[1%] are the respective significance levels.

We deal with the nonlinearity, nonstationary, and asymmetry attributable to the return series in a non-parametric fashion. We follow an embryonic decomposition technique with a robust transfer entropy approach, as discussed below.

### I-CEEMDAN

The latest strand of the EMD family, the I-CEEMDAN from [32], caters for noise that usually dominates short term [12, 33, 34]. Alternative methods include time-frequency approaches using wavelet methods, connectedness approach [35–38]. Its strengths include efficiency, the noise-to-signal ratio (SNR) compression of modal decompositions in dynamic signals, and precision with reconstruction [39, 40]. The I-CEEMDAN strand of [7], which has the best of these properties, is summarized as follows:

Stage I: Generate a new series by appending white noise *τ*_1_[*ω*^(*i*)^] to a signal *α*

$$\begin{array}{c} \alpha^{\left(i\right)}=\alpha +\rho_{0}(\omega^{\left(i\right)}),\;i=1,\;2,\;\ldots ,\;N, \end{array}$$ (1)

where *ω*^(*i*)^ is the *i*-th white noise term added, *ρ*_0_ denotes the SNR, and *N*. represents the number of added white noises.

Stage II: Estimate the local average of *α*^(*i*)^ by applying EMD to glean the opening residual

$$\begin{array}{c} r_{1}=(\frac{1}{N})\sum_{i=1}^{N}M(\alpha^{\left(i\right)}), \end{array}$$ (2)

from which first IMF *c*_1_ = *α* − *r*_1_ could be deduced.

Stage III: In a recursive process, generate the *k*-th IMF *c*_*k*_ = *r*_*k*−1_ − *r*_*k*_, for *k* ≥ 2, where

$$\begin{array}{c} r_{k}=(\frac{1}{N})\sum_{i=1}^{N}M(r_{k-1}+\rho_{k-1}\tau_{k}(\omega^{\left(i\right)})). \end{array}$$ (3)

### Transfer entropy (TE)

Let *I*, with marginal probability *p*(*i*), and *J*, with marginal probability p(*j*), represent two discrete random time series. Their joint probability is then defined as *p*(*i*, *j*). In order *k* (*process I*) and *I* (*process J*), we also assume dynamic stationarity for the Markov process. As stated by the Markov property, the probability at which *I* is observed in state *i* and time *t*+ 1 conditioned on *k* preceding data points is

$$\begin{array}{c} p(i_{t+1}|i_{t},\;\ldots ,\;i_{t-k+1})=\;p(i_{t+1}|i_{t},\;\ldots ,\;i_{t-k}). \end{array}$$ (4)

The mean bits needed for encoding the data point at *t* + 1 after knowing *k* observations is given as

$$\begin{array}{c} h_{j}\left(k\right)=-\sum_{i}^{}p\left(i_{t+1},\;i_{t}^{\left(k\right)}\right){\text{log}}_{2}\;p(i_{t+1}|i_{t}^{\left(k\right)}) \end{array}$$ (5)

where $i_{t}^{\left(k\right)}=(i_{t},\ldots ,\;i_{t-k+1}$) (correspondingly for process *J*).

The information flow to *I* from *J* is examined in a bivariate case by quantifying the variance from the Markov property $p(i_{t+1}|i_{t}^{\left(k\right)})=\;p(i_{t+1}|i_{t}^{\left(k\right)},j_{t}^{\left(I\right)})$.

Shannon’s entropy (SE) is then expressed as follows:

$$\begin{array}{c} T_{J⟶I}\left(k,l\right)=\sum_{}^{}P\left(i_{t+1},\;i_{t}^{\left(k\right)},\;j_{t}^{\left(I\right)}\right)log\frac{P(i_{t+1}|i_{t}^{\left(k\right)},\;j_{t}^{\left(I\right)})}{P(i_{t+1}|i_{t}^{\left(k\right)})}, \end{array}$$ (6)

where *T*_*J*→*I*_ aggregates the information flow toward *I* from *J*. Analogously, the flow of information toward *J* from *I*, which is *T*_*I*→*J*_, can be obtained. The net estimate of the information flow is computed as the excess of *T*_*J*→*I*_ over *T*_*I*→*J*_, which serves as the central information flow path.

The expediency of SE in the area of finance cannot be overemphasized, but it does not attribute equal weights to all probable expectations in a probability distribution. Note that fat tails are pervasive in asset pricing, but the SE does not overcome this assumption. Therefore, we resort to [41] transfer entropy (RTE), which uses a weighting value *q*, to overcome this shortfall of the SE. RTE is computed as

$$\begin{array}{c} H_{J}^{q}=\frac{1}{1-q}\;{\text{log}}_{2}\sum_{j}^{}P^{q}\left(j\right) \end{array}$$ (7)

with *q* > 0. For *q* → 1, RE and SE converge. For 0 < *q* < 1, more weight is assigned to low probability events, while for *q* > 1, outputs *j* with higher initial probabilities are favored by the weights. Resultantly, based on *q*, RTE facilitates the assignment of different weights to unequal regions of the distribution [13, 42, 43]. This feature of RTE makes it superior to SE and, hence, contributes to its desirability in finance.

The “escort distribution” ${⌀}_{q}\left(j\right)=\frac{p^{q}\left(j\right)}{\sum_{j}^{}p^{q}\left(j\right)}$ for *q* > 0 is applied to normalize the weighted distributions [44], from which RE is estimated as

$$\begin{array}{c} {RT}_{J⟶I}\left(k,l\right)=\frac{1}{1-q}p\left(i_{t+1},\;i_{t}^{\left(k\right)},\;j_{t}^{\left(I\right)}\right)\;{\text{log}}_{2}\frac{\sum_{i}^{}{⌀}_{q}(i_{t}^{\left(k\right)})P^{q}(i_{t+1}|i_{t}^{\left(k\right)})}{\sum_{i,j}^{}{⌀}_{q}(i_{t}^{\left(k\right)},j_{t}^{\left(I\right)})P^{q}(i_{t+1}|i_{t}^{\left(k\right)},\;j_{t}^{\left(I\right)})} \end{array}$$ (8)

Note that negative estimates could be provided by the RTE. Acknowledging *J*’s record, in this case, connotes significantly more uncertainty than acknowledging only *I*’s record would imply.

TE estimations are subject to biases in small samples [45]; the effective transfer entropy can resolve this and is derived as

$$\begin{array}{c} {ETE}_{J⟶I}\left(k,l\right)=T_{J⟶I}\left(k,l\right)-T_{Jshuffled⟶I}\left(k,l\right), \end{array}$$ (9)

where the TE using faltered forms of the data series *J* is represented as *T*_*Jshuffled*→*I*_(*k*, *l*). The procedure removes the serial reliance of the data series of *J*, while the statistical linkages between *J* and *I*are preserved through repetitive random draws from the given return series *J* and rearranging them to produce a fresh return series. We utilize the package “RTransferEntropy” in R programming.

## Empirical results and discussion

In this section, we present and discuss the empirical findings. We note that geopolitical risk (GPR) is driven by shocks to the international order. These shocks include, among others, wars, military actions, and diplomatic conflicts. As these events are predominantly political in nature, financial markets typically have no direct influence over them [6]. It would certainly be contrary to economic intuition to consider financial markets as contributors to geopolitical risk. By contrast, the impact of geopolitical shocks on financial markets is significant. Shocks emanating from geopolitical crises affect financial markets through risk transmission and contagion [28]. Therefore, in our analysis, we resort to the flow from geopolitical risk, as measured by GPR, to gauge the impact of geopolitical shocks on asset returns. This position is consistent with [12, 46, 47], who analyze the one-way flow from COVID-19 shocks to financial markets.

We focus on the one-way information flow from geopolitical risk (GPR) to major European assets and global commodities. The Rényian TE approach yields both high-risk (negative) and low-risk (positive) ETEs. In the spirit of risk management, we analyze the ETEs in line with portfolio diversification and the efficiency of these assets in the wake of geopolitical risk. Following the extant literature [12, 47, 48], we specify a fault weight of 0.30 by way of accounting for the return series’ tailed observations. We evaluate the dynamic responses of various assets to geopolitical risk at multi scales. The ETEs are represented by black dots located in the red bars. The ends of the red bars represent 95% confidence interval bounds. Therefore, we reject H0 of “no information flow” for any confidence bounds that completely fall in one region (be it positive or negative). A given ETE lacks statistical significance for any overlapping confidence boundaries at the origin. Tables 2 and 3 provide the numerical estimates of the ETEs (see S1 Appendix).

**Fig 2:**
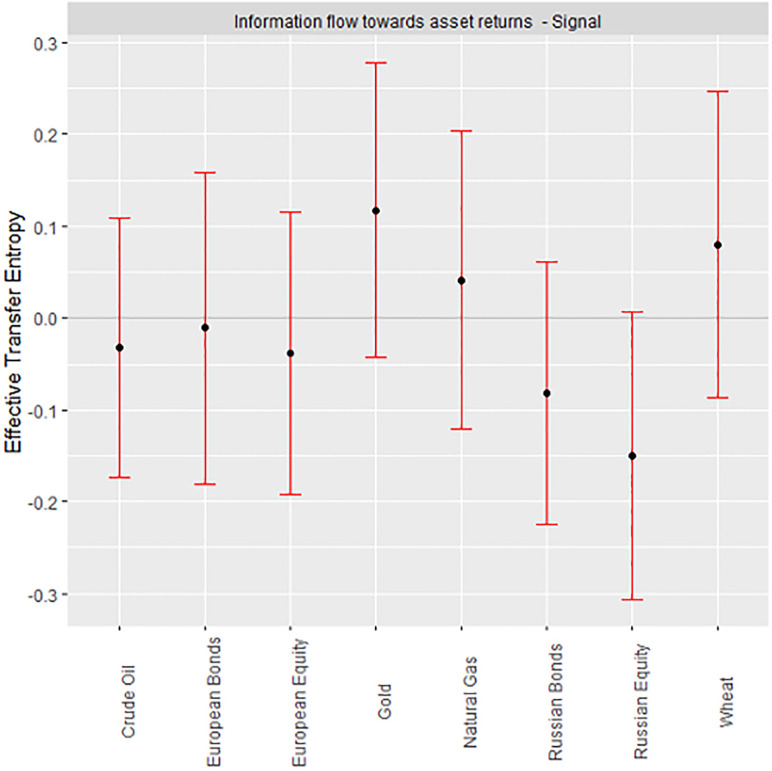
ETEs for signal.

For the signal data, ETEs are positive for all commodities save crude oil, while all traditional assets are negative ETE recipients (Fig 2). Nevertheless, significance cannot be attributed to any of the ETEs of the signal data. Signal ETEs imply that diversifying geopolitical risks may be insignificant despite the potential. By accounting for complexity, nonlinearity, and asymmetry in asset returns, the ETEs from the decomposed return series are examined.

**Fig 3:**
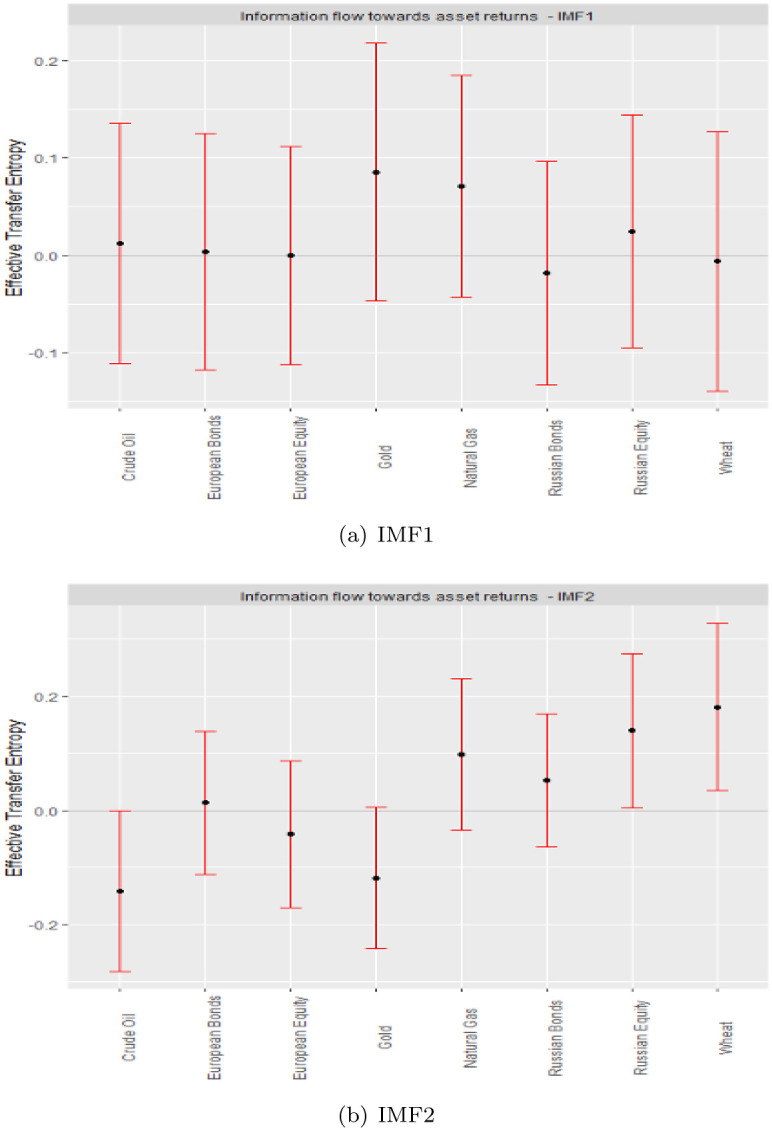
Short-term ETEs.

Following [12], IMF1 and 2 (Fig 3) are designated for the short term. ETEs in the short term suggest significantly high risks for crude oil returns but low-risk Russian equity and wheat. The high-and low-risk potentials of all other asset returns are insignificant in the short term. The information flow from geopolitical risk in the short term is distinctly received by various assets, as we identify negative and positive ETEs.

**Fig 4:**
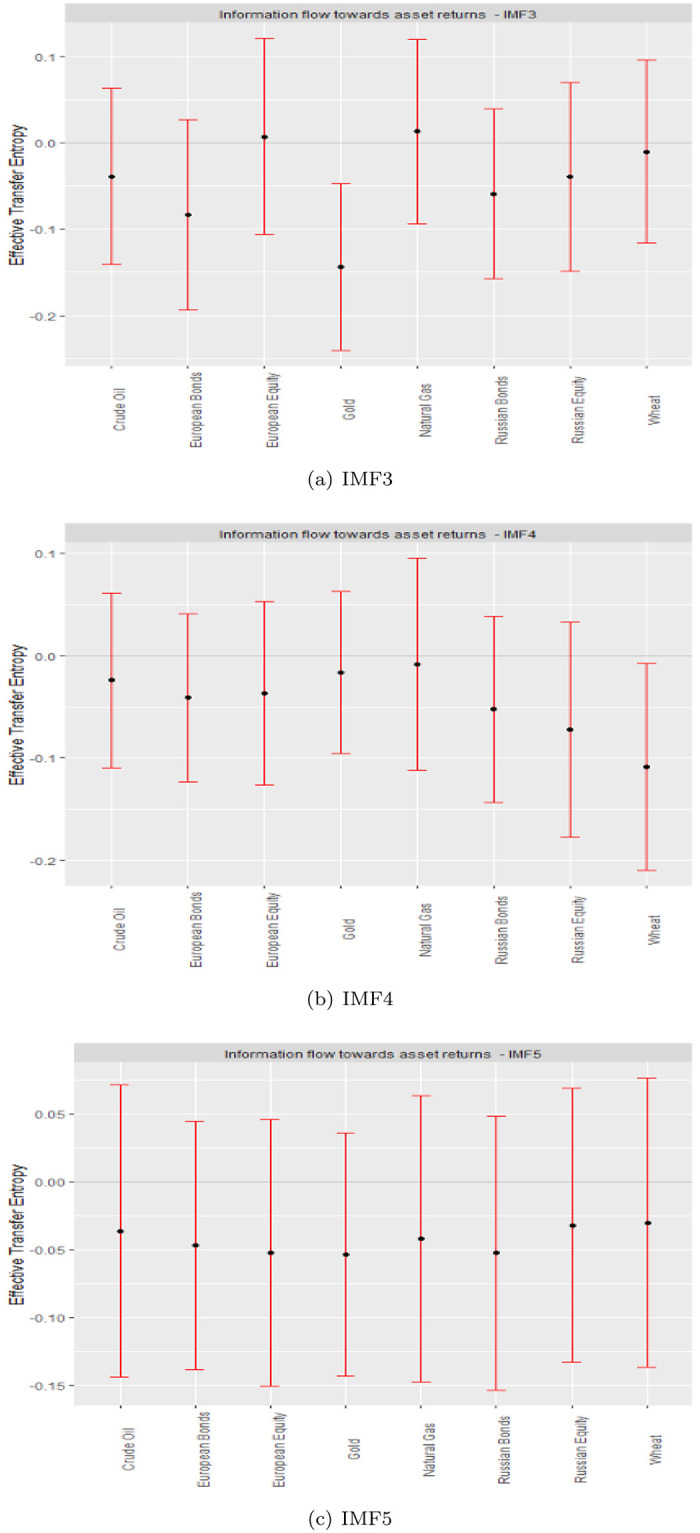
Medium-term ETEs.

The medium-term subtleties are indicated by IMFs 3 to 5 (Fig 4). It is worth noting that the number of high-risk (negative ETE receiving) assets increases with scales. Higher scales (IMFs) yield more negative ETEs, reflecting high uncertainties surrounding asset returns in medium- to long-term periods. Specifically, in the medium term, except for European equity and natural gas at IMF3, all assets receive negative information flow. Indicatively, the ETEs for gold and wheat prove significant at the medium scale.

**Fig 5:**
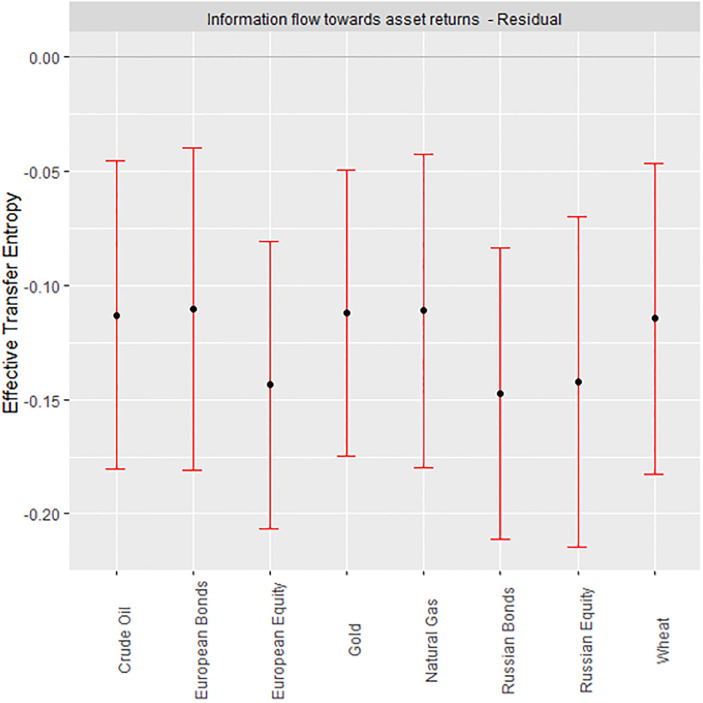
Long-term ETEs.

The fundamental characteristics of assets are noted to prevail in the long term. Thus, the IMF residual denotes long term (Fig 5). Long-term ETEs suggest significantly high risks for all assets. The information content transferred by geopolitical risk to the studied assets renders their returns highly risky. There is significantly more uncertainty when the history of geopolitical risk is incorporated into market returns from the studied assets.

Our findings have several implications. For the signal data, diversification prospects between high-risk and low-risk ETE recipients are insignificant, which we attribute to the existence of complexity in asset returns within the signal. In the presence of a complex, non-linear, and asymmetric market response, assets bear fat tails and noise that are accounted for in the I-CEEMDAN decomposition of the signal data into IMFs to represent various investment horizons and distinguish short-term dynamics from their medium-and long-term counterparts, an important parameter for investments [7, 12, 49]. Market participants’ attitudes symbolize the short term, the impact of key occurrences embodies the medium term, and fundamental linkages describe the long term [12, 34, 39, 50–52]. Our results imply diversification advantages across the short term, between the pairs of Russian equities and crude oil or wheat and crude oil at the short-term scale (IMF2).

The fact that the signal transfer entropies are insignificant, while those across the various scales bear some significance, emphasizes the need to explore the effect of GPR on various assets in a multiscale framework, hence, justifying the application of the I-CEEMDAN-based transfer entropy.

We find that market participants’ behavior varies across trading horizons, which can be inferred from multi-scale analyses. As markets respond rapidly to evolving events, investors modify their risk-return preferences to suit the prevailing conditions. Therefore, the effect of any geopolitical shock on financial markets will be manifested disparately by the various assets in these markets. Hence, in times of intense geopolitical risk, our findings call for market participants to understand how different assets react to GPR shocks across different investment scales. Policy actions by investors, portfolio managers, or regulators intended to manage risks or regulate markets must be reassessed across the short-, medium-, and long-term horizons by incorporating all available market information.

Our findings corroborate those of the existing studies that highlight variations in the impact of GPR and investor sentiment on various financial assets or markets [29–31, 53]. Our study emphasizes the opportunity for short-term diversification offered by these variations, which result from the information flow from GPR to the analyzed assets. Despite this, the efficiency of financial markets can be confirmed on a long-term scale, where various assets tend to bear similar responses to GPR.

## Conclusion

In this study, we investigate information flows from GPR caused by the Russian-Ukrainian conflict to various financial assets by combining the Rényi transfer entropy and I-CEEMDAN methods. Furthermore, we estimate the dynamic response of financial assets to GPR at multi-term scales, that is, short-, middle-, and long-term scales.

Our main findings are summarized as follows: First, in the short term, crude oil and Russian equity have positive and negative ETE values, respectively, corresponding to the GPR. Therefore, they can be a pair capable of exhibiting a portfolio diversification effect corresponding to GPR in the short term. Second, in the medium term, most assets have negative ETE values. In other words, information on GPR increases the risk of financial assets. This result is also observed in the long term. Third, efficiency of the studied asset markets lies in the long term, all of which respond significantly negatively to information flow from geopolitical risks, eliminating all forms of hedging and diversification benefits between traditional assets and essential commodities.

These findings have several implications for market participants. For example, investors can establish effective investment strategies by leveraging the short-term relationship between GPR and financial assets, as shown by the results of this study. Furthermore, this relationship can be utilized for asset allocation, risk taking, and portfolio management. In addition, since GPR information increases the risks associated with financial assets in the medium- and long-term, portfolio managers need to take action to hedge against an increase in GPR that is forecasted for a period beyond the medium term. Consequently, policymakers should monitor the relationship between GPR and financial assets over different timescales to avoid market turmoil. Our findings suggest that if policymakers were to monitor the flow of market information over different time scales (short-, medium-, and long-term) they could prevent the kind of financial market disruptions caused by the Russia-Ukraine conflict.

Despite the resilience of our methodological framework, a time-varying perspective of the information flow from GPR to the analyzed markets is lacking. Hence, in future studies, an analysis of the time-varying information flow would be useful to identify the changes in the patterns of information spillovers during your sampling period. Additionally, future studies can consider extending our study to other financial markets, such as the equity and bond markets in the United States and China. Such studies would allow us to understand how the Russia-Ukraine conflict has impacted global equity and bond markets.

## Supporting information

S1 Appendix (PDF)
