# Correction: Information flow dynamics between geopolitical risk and major asset returns

**DOI:** 10.1371/journal.pone.0294959

**Published:** 2023-11-21

**Authors:** Zaghum Umar, Ahmed Bossman, Sun-Yong Choi, Xuan Vinh Vo

In the Funding section, there is missing information. The correct funding statement is ‘The work of S.-Y. Choi was supported by the National Research Foundation of Korea (NRF) grant funded by the Korean government (MSIT) (No. 2021R1F1A1046138). This research is partly funded by Institute of Business Research, University of Economics Ho Chi Minh City, Ho Chi Minh City, Vietnam. The funders had no role in study design, data collection and analysis, decision to publish, or preparation of the manuscript.’
