# Supplementary material for: Information flow dynamics between geopolitical risk and major asset returns
Source: PLoS One. 2023 Apr 25;18(4):e0284811. doi: 10.1371/journal.pone.0284811 (PMC10128946; doi:10.1371/journal.pone.0284811)
Supplement: S1 Appendix — (PDF) [file pone.0284811.s001.pdf]

Appendix

Table 2. Effective transfer entropies. Notes : β – estimates of effective transfer entropy; SE – standard error; *[10%], **[5%], and ***[1%] are the respective significance levels of test statistics.

	Signal			IMF1			IMF2		
Flow	β	SE	t-stats	β	SE	t-stats	β	SE	t-stats
GPR → Crude Oil	-0.032	0.086	-0.376	0.012	0.075	0.161	-0.141*	0.086	-1.649
GPR → European Bonds	-0.011	0.103	-0.104	0.004	0.074	0.050	0.013	0.076	0.176
GPR → European Equity	-0.038	0.093	-0.407	0.000	0.068 -	0.005	-0.041	0.078	-0.529
GPR → Gold	0.117	0.098	1.201	0.086	0.080	1.066	-0.118	0.075	-1.567
GPR → Natural Gas	0.041	0.098	0.420	0.071	0.069	1.029	0.098	0.081	1.215
GPR → Russian Bonds	-0.082	0.087	-0.944	-0.018	0.070	-0.259	0.052	0.071	0.739
GPR → Russian Equity	-0.150	0.095	-1.579	0.024	0.073	0.337	0.139*	0.082	1.689
GPR → Wheat	0.080	0.102	0.787	-0.006	0.081	-0.076	0.181*	0.089	2.028

Table 3. Effective transfer entropies. Notes : β – estimates of effective transfer entropy; SE – standard error; *[10%], **[5%], and ***[1%] are the respective significance levels of test statistics.

	IMF3			IMF4			IMF5			Residual		
Flow	β	SE	t-stats	β	SE	t-stats	β	SE	t-stats	β	SE	t-stats
GPR → Crude Oil	-0.039	0.062	-0.626	-0.024	0.052	-0.461	-0.036	0.065	-0.556	-0.113***	0.041	-2.751
GPR → European Bonds	-0.083	0.067	-1.241	-0.041	0.050	-0.820	-0.047	0.055	-0.843	-0.110**	0.043	-2.568
GPR → European Equity	0.007	0.069	0.106	-0.037	0.055	-0.668	-0.052	0.060	-0.876	-0.144***	0.038	-3.753
GPR → Gold	-0.144**	0.059	-2.441	-0.016	0.048	-0.339	-0.053	0.054	-0.984	-0.112***	0.038	-2.939
GPR → Natural Gas	0.013	0.065	0.202	-0.008	0.063	-0.132	-0.042	0.064	-0.652	-0.111***	0.042	-2.663
GPR → Russian Bonds	-0.059	0.060	-0.988	-0.052	0.056	-0.943	-0.052	0.061	-0.855	-0.147***	0.039	-3.811
GPR → Russian Equity	-0.039	0.067	-0.586	-0.072	0.064	-1.124	-0.032	0.061	-0.521	-0.142***	0.044	-3.236
GPR → Wheat	-0.010	0.065	-0.153	-0.109	0.062	-1.766	-0.030	0.065	-0.466	-0.115***	0.041	-2.760
